# Association between SEMA3A signaling pathway genes and BMD/OP risk: An epidemiological and experimental study

**DOI:** 10.3389/fendo.2022.1014431

**Published:** 2022-11-08

**Authors:** Hao-long Zhou, Mu-hong Wei, Dong-sheng Di, Ru-yi Zhang, Jian-li Zhang, Ting-ting Yuan, Qian Liu, Ting-ting Zhou, Qin Huang, Qi Wang

**Affiliations:** ^1^ Key Laboratory of Environment and Health, Ministry of Education & Ministry of Environmental Protection, Department of Epidemiology and Biostatistics, School of Public Health, Tongji Medical College, Huazhong University of Science and Technology, Wuhan, China; ^2^ Department of Rehabilitation Medicine, Union Hospital, Tongji Medical College, Huazhong University of Science and Technology, Wuhan, China

**Keywords:** semaphorin 3A signaling pathway, bone mineral density, osteoporosis, *NRP1*, *PLXNA2*

## Abstract

**Objective:**

This study aimed to explore the associations of genetic variants in the semaphorin 3A (SEMA3A) signaling pathway genes, including *SEMA3A*, *NRP1*, *PLXNA1*, *PLXNA2* and *PLXNA3* with osteoporosis (OP) risk and bone mineral density (BMD) in a Chinese Han older adult population.

**Study design and method:**

A two-stage design was adopted. Total of 47.8kb regions in the 5 genes were sequenced using targeted next-generation sequencing (NGS) technology in the discovery stage, and the discovered OP-related single nucleotide polymorphisms (SNPs) were further genotyped using improved multiple linkage detection reaction technique in the validation stage. Methods of ALP/TRAP staining, real-time fluorescent quantitative PCR, and cell proliferation and apoptosis assays were performed with MC3T3-E1 and RAW 264.7 cell lines to clarify biological effects of observed functional variants in cell lines responsible for bone mass remodeling.

**Results:**

Total of 400 postmenopausal women (211 OP cases) were involved in the discovery stage, where 6 common and 4 rare genetic variants were found to be associated with OP risk. In the validation stage among another 859 participants (417 women, 270 OP cases), the *PLXNA2* rs2274446 T allele was associated with reduced OP risk and increased femoral neck (FN) BMD compared to the C allele. Moreover, significant associations of *NRP1* rs2070296 with FN BMD/OP risk and of *NRP1* rs180868035 with lumbar spine and FN BMDs were also observed in the combination dataset analysis. Compared to the osteoblasts/osteoclasts transfected with the wild-type *NRP1* rs180868035, those transfected with the mutant-type had reduced mRNA expression of osteoblastic genes (i.e., *ALP, RUNX2*, *SP7* and *OCN*), while elevated mRNA expression of osteoclastic genes (i.e., *TRAP*, *NFATc1* and *CTSK*). Furthermore, mutant *NRP1* rs180868035 transfection inhibited osteoblast proliferation and osteoclast apoptosis, while promoted osteoclast proliferation and osteoblast apoptosis in corresponding cell lines.

**Conclusion:**

Genetic variants located in *NRP1* and *PLXNA2* genes were associated with OP risk and BMD. The *NRP1* rs180868035 affects bone metabolism by influencing osteoblasts and osteoclasts differentiation, proliferation and apoptosis.

## 1 Introduction

Osteoporosis (OP) is a systemic skeletal disease characterized by low bone mineral density (BMD) and deteriorative bone microstructure. Fragility fracture is the most common and harmful complication of OP (1). Dual-energy X-ray absorptiometry (DXA)-derived BMD measurements at skeletal sites of total hip, lumbar spine (LS), and femoral neck (FN) were recommended for the diagnosis of OP (2). A systematic review and meta-analysis study demonstrated that the prevalence of OP was 18.3% worldwide, with a significantly higher prevalence in women (23.1%) than in men (11.7%) (3). It was reported the number of fragility fractures was estimated at 3.5 million in 2010, and it would increase 28%, from 3.5 million to 4.5 million, by 2025 in Europe (4). In a study involving 3157 American participants aged ≥50 years, 30% men and 49% women were diagnosed with osteopenia, and 2% men and 10% women were diagnosed with OP based on the BMD measurements at the FN (5). A recent nationwide epidemiological study in China estimated the age-standardized prevalence of OP was 6.46% and 29.13% for men and women aged ≥50 years, corresponding to 10.9 million men and 49.3 million women (6). Above-mentioned evidence suggests that the prevalence of OP stands at a high level, and OP not only causes high disability and mortality, but brings a heavy burden to society, becoming a serious public health issue.

Bone remodeling is a dynamically balanced process regulated by both osteoblasts and osteoclasts. OP occurs when osteoclast-induced bone mass loss exceeds osteoblast-induced bone mass formation. Semaphorin 3A (SEMA3A) is one of the semaphorin family members that are involved in various physiological and pathological activities in human body, e.g., embryonic and nervous system development, immune regulation, promotion of inflammation reduction and oncogenesis (7–10). Recently, *SEMA3A* was also found to possess dual role of promoting osteoblastogenesis and inhibiting osteoclastogenesis, suggesting that it may be a potential therapeutic target for OP (11). Animal studies found that the SEMA3A supplementation *via* transcutaneous injection into the center of the distraction zone elevated BMD, tissue mineral density (TMD) and vascular density of operative area (of tibia) in adult male mice underwent tibia osteotomy surgery (12). Hayashi et al. observed obvious OP phenotype in *SEMA3A* gene knocked out mice (13). Liu et al. found that fasting serum SEMA3A was positively correlated with bone formation marker—osteocalcin (OCN), while no significant association was found of fasting serum SEMA3A with BMD/osteoporotic fractures (14).

Neuropilin-1 (NRP1) is a type I transmembrane protein which possesses high affinity to SEMA3A, and it plays an indispensable role in the chemorepulsion mediated by SMEA3A (15). PlexinA, one class of the plexin proteins, is the major receptors for SEMA3A. SEMA3A binds to NRP1 to assemble and to active NRP-plexinA holoreceptor complexes (16). It was found that the *NRP1*-mutant mice exhibited a same osteoporotic phenotype as the *SEMA3A*-deficient mice, suggesting that the SEMA3A function through NRP1 (13). SEMA3A enhanced the competitive binding of NRP1 (against TREM2) with plexinA1 (PLXNA1), thereby blocking the PLXNA1-TREM2-DAP12 complex formation to inhibit osteoclast differentiation (13). Otherwise, SEMA3A can also bind to the NRP1-PLXNA1 receptor complex to promote osteoblast formation (17). *In vitro*, plexinA2 (PLXNA2) were found to influence the expression of RUNX2 and SP7—two major osteogenic transcription factors, further regulating osteogenic differentiation and mineralization (18). PlexinA3 (PLXNA3) may participate in the process of SEMA3A regulating bone innervation (19).

Taking all evidence above, the SEMA3A signaling pathway may be involved in the OP pathology, and relevant genes may be associated with the disease susceptibility. However, epidemiological and experimental evidence is still scarce. Herein, we conducted a two-stage study in 1259 Chinese Han elderly to explore OP-related genetic variants in SEMA3A signaling pathway genes, including *SEMA3A, NRP1*, *PLXNA1*, *PLXNA2* and *PLXNA3*, using next generation sequencing (NGS) and improved multiple linkage detection reaction (iMLDR) technique. And *in vitro* experiments were also conducted to explore potential functions and biological mechanisms of revealed OP-related genetic loci.

## 2 Method and materials

### 2.1 Study design and participants

**Figure 1:**
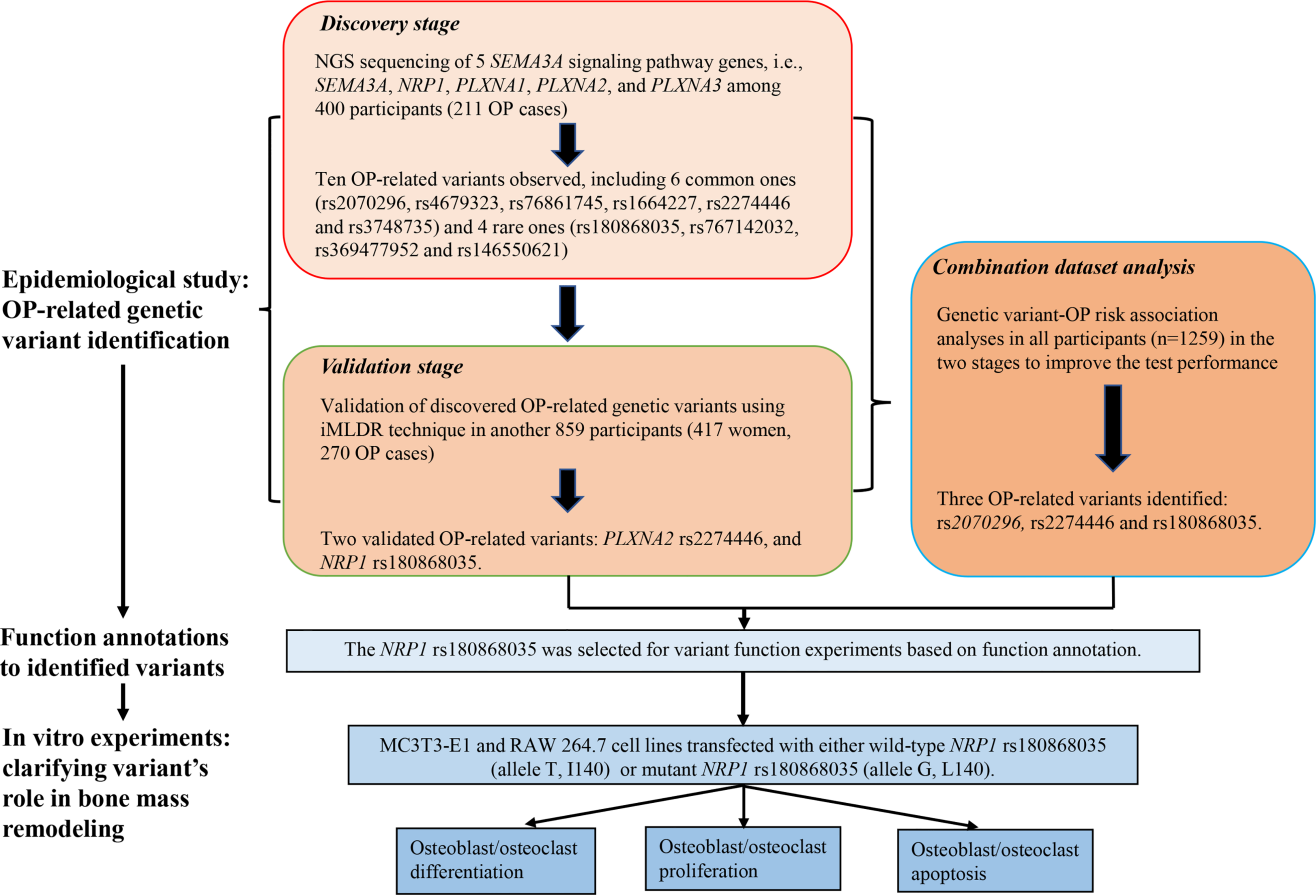
Framework of the study design.

The work of this study consisted of four parts. First, we conducted NGS of 5 candidate genes (*SEMA3A*, *NRP1*, *PLXNA1*, *PLXNA2*, and *PLXNA3*) using Illumina Hiseq 2500 sequencing platform based on microarray capture sequencing method to discover OP-related genetic variants in 400 Chinese postmenopausal women (211 OP cases). Second, the discovered OP-related single nucleotide polymorphisms (SNPs) were further genotyped using iMLDR technique in another 859 Chinese participants (417 women, 270 OP cases) in the validation stage. Third, we conducted a combination dataset analysis of genotyping data in all participants in the discovery and validation stages to improve the test performance. After the three steps for OP-related genetic variant identification, bioinformatic tools were adopted for function annotations to the identified variants. Fourth, *in vitro* experiments were conducted to clarify the roles of disease-related and functional genetic variants in bone mass remodeling. Considering that postmenopausal women is a high-risk group for OP, we included postmenopausal women exclusively in the discovery stage to identify as many OP-related genetic variants as possible. Detailed illustration to the study design is shown in **Figure 1**.

Participants were recruited from two communities in Wuhan and physical examination and rehabilitation center of Wuhan Union hospital during 2017–2018. The trained staff conducted an interview by phone to determine whether these participants were eligible for this study. We included Chinese Han participants aged ≥60 years old. We excluded participants: (i) with previous hysterectomy, ovarian and adnexal resection; (ii) with other prevalent endocrine diseases that may potentially influenced bone metabolism (i.e., hyperthyroidism and hypothyroidism); (iii) with hereditary bone disease, including osteogenesis imperfecta, osteochondrosis and multiple myeloma; (iv) who ever took hormonal medicines for a long time (>3 months) or in the past 6 months; and (v) with unavailable data on candidate genes and BMD. Finally, a total of 1259 participants were involved in this study. OP diagnosis is defined as the T value ≤-2.5 (BMD values is ≤2.5 standard deviation below the BMD values in normal adult with same sex and race) based on the World Health Organization OP diagnostic criteria (2). Thus, those passed the inclusion and exclusion criteria and met OP diagnostic criteria were assigned to the OP group, and the rest were assigned to the non-OP group.

This study was approved by the Ethics Committee of Tongji Medical College of Huazhong University of Science and Technology. Written informed consent was obtained from each participant before enrollment.

### 2.2 Data collection and BMD measurement

Questionnaire survey was conducted to collect information on general characteristics (name, gender and age), medical history (fracture, uterus and ovaries surgery and thyroid-related disease), medication history (hormones), lifestyle (smoking and drinking), and menopause status for women only (menarche age and menopause age) from each participant. Body mass index (BMI) was obtained from physical examination, and it was calculated as weight (kg) divided by the square of height (m). BMD at the skeletal sites of the LS and FN was measured by Dual-energy X-ray absorptiometry (Lunar Prodigy, GE, USA).

### 2.3 Genotyping and function annotation

Before the physical examination, 3 mL venous blood was drawn from each participant for genotyping. The Relax Gene Blood DNA System Kit (Tiangen, Beijing, China) was adopted to extract genomic DNA. The NGS was used to screen for genetic variants that were potentially associated with OP in the discovery stage. IMLDR technique was used for genotyping the discovered OP-related SNPs in the validation stage. During this process, we set negative controls for each plate, and 5% of double-blind samples were randomly selected for repeat typing verification. The consistency of typing results was 100%, indicating the reliability of iMLDR technique.

Based on minor allele frequency (MAF), genetic variants were classified into common variants (MAF≥0.01) and rare variants (MAF<0.01). The Sorting Intolerant From Tolerant (SIFT) (http://sift.jcvi.org/www/SIFT_chr_coords_submit.html), Polyphen2 (http://genetics.bwh.harvard.edu/pph/pph_help_text.html), and Mutation Taster software (http://sites.google.com/site/jpopgen/dbNSFP) were adopted to predict deleteriousness of mutations.

### 2.4 *In vitro* experiments

#### 2.4.1 Plasmid construction and lentivirus packaging

Sequence of *NRP1* gene was obtained from the NCBI website. The cDNA sequence of wild-type *NRP1* (allele T at rs180868035, I140) and mutant *NRP1* (allele G at rs180868035, L140) were inserted into the vector plasmid, named pLVX-NRP1-Puro and pLVX-NRP1-Mut-Puro, respectively. The correctness of the inserted target fragment was verified by sequencing. Both plasmid construction and validation were done by Wuhan Vapol Biological company. The pLVX-NRP1-Puro and pLVX-NRP1-Mut-Puro were introduced into 293 T cells by lentiviral packaging to produce high titer lentivirus containing the target fragment.

#### 2.4.2 Construction of MC3T3-E1 and RAW 264.7 stable-transfer cell lines

MC3T3-E1 and RAW 264.7 cell lines were used as models for osteoblast and osteoclast, respectively. Both cell lines were purchased from Wuhan Procell Life Science & Technology Co., Ltd. Construction of each stable-transfer cell line included 5 steps: (1) cell recovery and culture: α-MEM+10%FBS+1% (Penicillin-Streptomycin Solution) and DMEM+10%FBS+1% (Penicillin-Streptomycin Solution) were suitable culture medium for MC3T3-E1 and RAW 264.7 cell lines, separately; (2) cell passaging; (3) determine the optimal screening concentration of puromycin; (4) lentiviral infection; and (5) cell screening.

#### 2.4.3 Measurement of osteoblast differentiation ability

Alkaline phosphatase (ALP) staining test was used to measure the activity of ALP, which was reckoned as the hallmark enzyme of mature osteoblast. The HiScript II Q Select RT SuperMix for qPCR (+ gDNA wiper) kit was used to reverse transcribe RNA into DNA, and real-time fluorescent quantitative PCR was performed to detect the mRNA expression levels of osteoblast marker genes, including *ALP*, *RUNX2*, *SP7* and *osteocalcin* (*OCN*). In the process, the cDNA synthesized by reverse transcription, as a template, was amplified on an ABI QuantStudio 6 real-rime fluorescence quantitative PCR instrument using SYBR Green Master Mix reagent. The *GAPDH* gene was used as an internal reference for the relative quantification of osteoblast marker genes mRNA levels, and the relative quantification was calculated using the 2^-ΔΔCt^ method.

#### 2.4.4 Measurement of osteoclast differentiation ability

Tartrate-resistant acid phosphatase (TRAP) staining test was used to measure the activity of TRAP, which was reckoned as the hallmark enzyme of mature osteoclast. The HiScript II Q Select RT SuperMix for qPCR (+ gDNA wiper) kit was used to reverse transcribe RNA into DNA, and real-time fluorescent quantitative PCR was performed to detect the mRNA expression levels of osteoclast marker genes, including *TRAP*, *nuclear factor of activated T cells c1* (*NFATc1*) and *cathepsin K* (*CTSK*). Other processes were consistent with above.

#### 2.4.5 Cell proliferation assay

The proliferation ability of osteoblast and osteoclast was detected by CCK8 cell proliferation and cytotoxicity assay kit. MC3T3-E1 and RAW 264.7 cells in good logarithmic growth phase were inoculated into 96-well plates at a concentration of 5×10^3^ and 2×10^3^ cells, respectively, per well, and three replicate wells were set up for each group. Then they were incubated at 37 °C in a 5% CO_2_ incubator for 48 hours. And 10 μL CCK8 solution was added to each well and incubated for 4 hours at 37 °C, then the optical density (OD) value at 450 nm of each well was measured by enzyme-labeled instrument.

#### 2.4.6 Cell apoptosis assay

The AnnexinV-APC/7-AAD apoptosis detection kit was adopted for apoptosis assay. The kit uses fluorescein APC-labeled Annexin V as a probe to reflect early apoptosis by binding phosphatidylserine exposed on the outside of the cell to the cytosol of early apoptosis cells. The 7-AAD, provided in this kit, is a nucleic acid dye that cannot penetrate the intact cell membrane of normal or early-stage apoptotic cells, but it can penetrate and bind to DNA within late-stage apoptotic or necrotic cells. Thus, it can be used to distinguish surviving early-stage cells from necrotic or late-stage apoptotic cells. When Annexin V-APC was used in combination with 7-AAD, 7-AAD was excluded from live cells and early apoptotic cells, while late apoptotic cells and necrotic cells were stained, presenting double positive status.

### 2.5 Statistical analyses

Continuous variables were presented as mean ± standard deviation (SD) or median (interquartile range, IQR) where appropriate, and Student’s t test or Mann-Whitney U test was used to exam between-group difference. Categorical variables were presented as number (percentage), and Pearson χ^2^ test was adopted to exam difference between two groups. The χ^2^ Goodness-of -Fit test was applied to assess whether the genotype distribution of genetic variants in the control group conformed to the Hardy-Weinberg Equilibrium (HWE). The associations between common variants and BMD/OP risk were analyzed using four kinds of multiple linear regression models or multivariate-adjusted logistic regression models, including co-dominant, additive, dominant and recessive models. For rare variants, considering the small sample size of control group, the East Asian population in the GnomAD database was also used as the control. Fisher’s exact test was adopted to compare the allele frequency and genotype distribution of rare variants between OP and non-OP groups, and linear regression was used to analyze the association of rare variants with BMD. The discovery stage was designed to investigate potentially OP-related genetic variant therefore the results of this stage were not corrected for false discovery rate (FDR). The other results were corrected for FDR to control the false positive rate caused by multiple comparisons. Statistical analyses were performed using IBM SPSS v22.0 and R v4.0.3 (R Core Team, Vienna, Austria), and picture production was achieved by GraphPad Prism v5.01. All statistical analyses were two-sided, and a *P* value < 0.05 indicated statistical significance.

## 3 Results

### 3.1 General characteristics

General characteristics of all participants were presented in **Table 1**. In the discovery stage involving 400 postmenopausal women (211 OP cases), the mean (SD) age of OP cases was 67.67 (5.87) years, comparatively larger than the non-OP individuals aged 66.26 years (4.63) (*P*=0.008). The mean (SD) BMI of OP cases was 23.22 (3.14) kg/m^2^, significantly lower than the counterpart values (25.24 kg/m^2^ (SD: 3.05)) of non-OP group (*P*<0.001). The proportions of smoker and drinker in both OP and non-OP groups were no more than 3.3% and 2.4%, and both two proportions were not significantly different between two groups. The mean (SD) BMD measurement and T value at the skeletal sites of LS were 0.80 (0.08) g/cm^2^ and -3.12 (0.63), and were 0.66 (0.08) g/cm^2^ and -2.66 (0.67) of FN in OP group, significantly lower than the counterpart values, sequentially were 1.08 (0.15) g/cm^2^, -0.80 (1.09), 0.83 (0.09) g/cm^2^ and -1.25 (0.77), in non-OP group (all *P*<0.001).

**Table 1:** General characteristics of involved participants.

Variables		Discovery stage (n = 400)			Validation stage (n = 859)		
		OP	Non-OP	*P*	OP	Non-OP	*P*
n (%)							
Gender^&^	Women	211 (100)	189 (100)	–	164 (60.7)	253 (43.0)	**<0.001**
	Men	–	–		106 (39.3)	336 (57.0)	
Smoking^&^	Yes	7 (3.3)	3 (1.6)	0.433	43 (16.2)	101 (17.5)	0.624
	No	204 (96.1)	186 (98.4)		227 (83.8)	488 (82.5)	
Drinking^&^	Yes	5 (2.4)	0 (0)	0.093	37 (13.9)	107 (18.5)	0.099
	No	206 (97.6)	189 (100)		233 (86.1)	482 (81.5)	
Mean ± SD							
Age (years) ^#^		67.67 ± 5.87	66.26 ± 4.63	**0.008**	68.36 ± 6.02	67.36 ± 6.29	**0.029**
Height (cm) ^#^		153.05 ± 5.88	155.49 ± 5.50	**<0.001**	158.35 ± 7.50	161.93 ± 7.98	**<0.001**
Weight (kg) ^#^		54.36 ± 7.80	61.02 ± 7.96	**<0.001**	58.22 ± 9.93	64.76 ± 10.29	**<0.001**
BMI (kg/m^2^) ^#^		23.22 ± 3.14	25.24 ± 3.05	**<0.001**	23.15 ± 3.15	24.65 ± 3.14	**<0.001**
Age of menopause (years)^#^		48.85 ± 3.89	49.75 ± 4.32	**0.031**	48.72 ± 4.01	50.26 ± 3.38	**<0.001**
Age of menarche (years)^#^		14.02 ± 1.89	14.12 ± 1.88	0.614	14.01 ± 1.79	13.78 ± 1.81	0.231
LS BMD (g/cm^2^) ^#^		0.80 ± 0.08	1.08 ± 0.15	**<0.001**	0.89 ± 0.13	1.13 ± 0.18	**<0.001**
LS T Value ^#^		-3.12 ± 0.63	-0.80 ± 1.09	**<0.001**	-2.51 ± 1.03	-0.52 ± 1.43	**<0.001**
FN BMD (g/cm^2^) ^#^		0.66 ± 0.08	0.83 ± 0.09	**<0.001**	0.70 ± 0.16	0.85 ± 0.11	**<0.001**
FN T value ^#^		-2.66 ± 0.67	-1.25 ± 0.77	**<0.001**	-2.62 ± 0.69	-1.35 ± 0.81	**<0.001**

SD, standard deviation; BMI, body mass index; LS, lumbar spine; BMD, bone mineral density; FN, femoral neck; OP, osteoporosis.

^&^ χ^2^ test was used to compare difference between OP and non-OP groups; ^#^ T-test was used to compare difference between OP and non-OP group.

Boldness indicates the results achieve statistical significance (P<0.05).

In the validation stage involving 859 older adults (270 OP cases), the proportion of women was 60.7% in the OP group, significantly higher than that in the non-OP group (43.0%) (*P*<0.001). The mean (SD) age of OP cases was 68.36 years (6.02), higher than the counterpart values (67.36 years (6.29)) of the non-OP group (*P*=0.029). The OP individuals had lower BMI (mean of 23.15 kg/m^2^, SD of 3.15) than the non-OP individuals (mean of 24.65 kg/m^2^, SD of 3.14) (*P*<0.001). Nearly similar proportion of OP (16.2%) and non-OP (17.5%) individuals smoke, and more reported drinking in the latter (18.5%) than the former (13.9%) without significance though. The mean (SD) BMD and T values at the skeletal sites of LS were 0.89 g/cm^2^ (0.13) and -2.51 (1.03), and were 0.70 g/cm^2^ (0.16) and -2.62 (0.69) of FN in OP group, significantly lower than the counterpart values (1.13 (0.18) g/cm^2^ and -0.52 (1.43) at LS1-4, and 0.85 g/cm^2^ (0.11) and -1.35 (0.81) at FN) of non-OP group (all *P*<0.001).

### 3.2 Results in the discovery stage

A total of 794 genetic variants were genotyped in the 5 SEMA3A signaling pathway genes. After excluding genetic variants (i) with low quality and possible repetitive sequences, (ii) with average sequencing depth ≤30×, (iii) with detection rate ≤90%, and (iv) not met HWE equilibrium, 586 variants were retained for subsequent analysis. Among them, 119 variants were new, the rs numbers of whom were unavailable based on databases of 1000G, ExAC, ESP6500 and GnomAD. Among the newly found variants, 12 were nonsynonymous mutations locating in the *SEMA3A* (n=2), *NRP1* (n=1), *PLXNA1* (n=3), *PLXNA2* (n=3) and *PLXNA3* (n=3) genes, respectively. It was predicted that two of them were deleterious to protein function by at least two of three software i.e., the SIFT, POLYphen2 and MutationTaster (**Table S1**).

Results of associations between the sequenced common variants and OP risk are presented in **Table S2**. After adjusting for age, BMI and menopause age, 6 common variants were associated with OP risk, including *NRP1* rs2070296, *PLXNA1* rs4679323 and rs73861745, and *PLXNA2* rs1664227, rs2274446 and rs3748735. Compared to the wild genotype carriers (i.e., the rs4679323 CC, rs1664227 GG, rs2274446 CC and rs3748735 CC), carriers of rs4679323 CA (OR=0.53, 95% CI: 0.31, 0.90), rs2274446 CT (OR=0.55, 95% CI: 0.34, 0.88) and rs3748735 CT (OR=0.55, 95% CI: 0.32, 0.93) genotypes were less likely to have prevalent OP, while the rs1664227 CC genotype carriers were more likely to have prevalent OP (OR=2.04, 95% CI: 1.07, 3.86). Results of dominant, recessive and additive models were also presented (**Table S2**).

As shown in **Table S3**, the MAF distribution of four rare variants, i.e., the rs180868035 and rs767142032 in *NRP1*, and rs369477952 and rs146550621 in *PLXNA1* were found to be significantly different between OP group and the East Asian population in the GnomAD database, with *P* values of 0.019, 0.022, 0.005 and 0.022 sequentially (**Table S3**).

### 3.3 Results in the validation stage

Above-mentioned 10 OP-related genetic variants (6 common and 4 rare ones) were further genotyped in another 859 subjects in the validation stage. Characteristics of the 10 variants are shown in **Table S4**. Compared to the *NRP1* rs2070296 CC/CT genotype, the TT genotype was associated with an increased OP risk (OR=1.46, 95%CI: 1.01, 2.11), while the association did not achieve an FDR-corrected significance (**Table 2**). The *PLXNA2* rs2274446 CT/TT genotype carriers had a 37% reduction in OP risk (OR=0.63, 95%CI: 0.46, 0.87, *P_FDR_=*0.027) compared to the CC genotype carriers; and OP risk decreased by about 32% on average in response to a T to C mutant in the *PLXNA2* rs2274446 (OR=0.68, 95%CI: 0.52, 0.89, *P_FDR_=*0.029).

**Table 2:** Multivariate logistic regression results of associations between common genetic variants and OP risk in the validation stage.

Rs number	Genotype	OP	Non-OP	OR (95%CI)	*P* ^a^	*P*_FDR_ ^a^ ^b^
rs2070296	CC	74	174	1		
	CT	132	313	0.91 (0.64, 1.30)	0.619	0.743
	TT	64	102	1.38 (0.89, 2.12)	0.149	0.447
	Dominant model			1.03 (0.74, 1.44)	0.875	0.972
	Recessive model			1.46 (1.01, 2.11)	**0.045**	0.271
	Additive model			1.15 (0.93, 1.44)	0.206	0.618
rs4679323	CC	61	147	1		
	CA	142	289	1.12 (0.77, 1.64)	0.544	0.743
	AA	67	152	1.01 (0.65, 1.55)	0.978	0.978
	Dominant model			1.08 (0.76, 1.55)	0.660	0.972
	Recessive model			0.93 (0.66, 1.31)	0.677	0.892
	Additive model			1.00 (0.81, 1.24)	0.994	0.994
rs73861745	GG	147	321	1		
	GA	108	221	1.04 (0.76, 1.42)	0.827	0.827
	AA	15	46	0.84 (0.44, 1.59)	0.591	0.866
	Dominant model			1.01 (0.74, 1.36)	0.972	0.972
	Recessive model			0.83 (0.44, 1.55)	0.552	0.892
	Additive model			0.97 (0.76, 1.24)	0.833	0.994
rs1664227	GG	80	183	1		
	GC	134	283	1.17 (0.82, 1.66)	0.381	0.743
	CC	56	123	1.08 (0.70, 1.66)	0.721	0.866
	Dominant model			1.14 (0.82, 1.59)	0.429	0.859
	Recessive model			0.98 (0.68, 1.42)	0.926	0.926
	Additive model			1.05 (0.85, 1.30)	0.646	0.969
rs2274446	CC	187	345	1		
	CT	73	208	0.65 (0.46, 0.90)	**0.011**	0.063
	TT	10	37	0.53 (0.25, 1.12)	0.095	0.447
	Dominant model			0.63 (0.46, 0.87)	**0.005**	**0.027**
	Recessive model			0.61 (0.29, 1.28)	0.191	0.572
	Additive model			0.68 (0.52, 0.89)	**0.005**	**0.029**
rs3748735	CC	207	437	1		
	CT	59	141	0.86 (0.60, 1.23)	0.405	0.743
	TT	4	11	0.78 (0.23, 2.68)	0.698	0.866
	Dominant model			0.85 (0.60, 1.21)	0.377	0.859
	Recessive model			0.81 (0.24, 2.78)	0.743	0.892
	Additive model			0.87 (0.63, 1.19)	0.374	0.747

OP, osteoporosis; OR, odds ratio; CI, confidence interval.

a Covariates included age, body mass index and menopause age (for women only).

b P values corrected for false discovery rate.

Boldness indicates the results achieve statistical significance (P<0.05).

Results of associations of the 6 common variants and BMD measurements at skeletal sites of LS and FN are presented in **Table 3**. We found the *PLXNA2* rs2274446 CT/TT genotype was associated with increased FN BMD (β=0.030 g/cm^2^, 95%CI: 0.010, 0.050, *P_FDR_=*0.004) compared to the CC genotype, and the FN BMD increased by 0.030 g/cm^2^ (95% CI: 0.010, 0.040, *P_FDR_=*0.006) on average in response to a T allele increase.

**Table 3:** Multivariate linear regression results of associations between common genetic variants and BMDs in the validation stage.

SNPs	Models	LS BMD (g/cm^2^)			FN BMD (g/cm^2^)		
		β (95%CI)	*P* ^a^	*P*_FDR_ ^a^ ^b^	β (95%CI)	*P* ^a^	*P*_FDR_ ^a^ ^b^
rs2070296	Dominant model	-0.001 (-0.030, 0.020)	0.943	0.959	-0.001 (-0.020, 0.020)	0.891	1
	Recessive model	-0.010 (-0.040, 0.020)	0.422	0.785	-0.020 (-0.040, 0.001)	0.056	0.339
	Additive model	-0.004 (-0.020, 0.010)	0.611	0.821	-0.010 (-0.020, 0.010)	0.235	0.564
rs4679323	Dominant model	-0.001 (-0.030, 0.030)	0.959	0.959	0 (-0.020, 0.020)	1	1
	Recessive model	-0.004 (-0.030, 0.020)	0.753	0.825	-0.003 (-0.020, 0.020)	0.735	0.934
	Additive model	-0.002 (-0.020, 0.010)	0.821	0.821	-0.001 (-0.010, 0.010)	0.835	0.965
rs73861745	Dominant model	0.010 (-0.010, 0.030)	0.376	0.959	0.010 (-0.010, 0.030)	0.363	1
	Recessive model	0.030 (-0.010, 0.080)	0.127	0.765	0.020 (-0.020, 0.050)	0.391	0.782
	Additive model	0.010 (-0.010, 0.030)	0.184	0.551	0.010 (-0.010, 0.020)	0.282	0.564
rs1664227	Dominant model	-0.020 (-0.040, 0.010)	0.212	0.959	0.001 (-0.020, 0.020)	0.888	1
	Recessive model	-0.020 (-0.040, 0.010)	0.280	0.785	-0.020 (-0.040, 0.010)	0.934	0.934
	Additive model	-0.010 (-0.030, 0.004)	0.154	0.551	0.0003 (-0.010, 0.010)	0.965	0.965
rs2274446	Dominant model	0.010 (-0.020, 0.030)	0.646	0.959	0.030 (0.010, 0.050)	**<0.001**	**0.004**
	Recessive model	-0.010 (-0.060, 0.040)	0.825	0.825	0.030 (-0.010, 0.060)	0.190	0.571
	Additive model	0.003 (-0.020, 0.020)	0.771	0.821	0.030 (0.010, 0.040)	**<0.001**	**0.006**
rs3748735	Dominant model	-0.003 (-0.030, 0.020)	0.838	0.959	-0.004 (-0.020, 0.020)	0.691	1
	Recessive model	-0.030 (-0.110, 0.060)	0.523	0.785	-0.010 (-0.080, 0.060)	0.796	0.934
	Additive model	-0.004 (-0.030, 0.020)	0.720	0.821	-0.004 (-0.020, 0.010)	0.668	0.965

LS, lumbar spine; BMD, bone mineral density; FN, femoral neck; β, regression coefficient; CI, confidence interval.

a Covariates included age, body mass index and age of menopause (for women only).

b P values corrected for false discovery rate.

Boldness indicates the results achieve statistical significance (P<0.05).

As to the rare variant-BMD association, it was found that the BMD decreased in response to per rs180868035 G allele mutation at skeletal sites of LS (β=-0.282 g/cm^2^, 95%CI: -0.507, -0.057) and FN (β=-0.208 g/cm^2^, 95%CI: -0.375, -0.035), respectively.

### 3.4 Results in combination dataset analysis

In general, the significant associations found in the combination dataset analysis were consistent with those found in the validation stage. Notably, we also found the *NRP1* rs2070296 TT genotype was associated with higher OP risk (OR=1.63, 95%CI: 1.21, 2.19, *P_FDR_=*0.007) and lower FN BMD (β=-0.030 g/cm^2^, 95% CI: -0.050, -0.010, *P_FDR_=*0.004) compared to the CC/CT genotype (**Tables 4**–**6**).

**Table 4:** Multivariate logistic regression results of associations between common genetic variants and OP risk in the combination dataset analysis.

Rs number	Genotype	OP	Non-OP		OR (95%CI)		*P* ^a^		*P*_FDR_ ^a^ ^b^
rs2070296	CC	124	224		1				
	CT	229	417		0.91 (0.68, 1.22)		0.538		0.645
	TT	126	137		1.53 (1.08, 2.18)		**0.016**		0.098
	Dominant model				1.07 (0.81, 1.40)		0.648		0.777
	Recessive model				1.63 (1.21, 2.19)		**0.001**		**0.007**
	Additive model				1.22 (1.02, 1.46)		**0.027**		0.080
rs4679323	CC	124	191		1				
	CA	241	392		0.89 (0.66, 1.19)		0.421		0.631
	AA	110	194		0.85 (0.60, 1.21)		0.365		0.548
	Dominant model				0.88 (0.66, 1.16)		0.348		0.521
	Recessive model				0.92 (0.70, 1.23)		0.587		0.705
	Additive model				0.92 (0.78, 1.10)		0.362		0.434
rs73861745	GG	260	431		1				
	GA	177	290		0.98 (0.75, 1.26)		0.852		0.852
	AA	32	54		1.15 (0.70, 1.89)		0.582		0.699
	Dominant model				1.0 (0.78, 1.28)		0.998		0.998
	Recessive model					1.16 (0.72, 1.88)		0.547	0.705
	Additive model				1.03 (0.84, 1.25)		0.808		0.808
rs1664227	GG	132	250		1				
	GC	244	374		1.28 (0.96, 1.69)		0.089		0.179
	CC	103	154		1.32 (0.94, 1.88)		0.114		0.341
	Dominant model				1.29 (0.99, 1.69)		0.060		0.143
	Recessive model				1.14 (0.84, 1.53)		0.400		0.705
	Additive model				1.16 (0.98, 1.38)		0.088		0.143
rs2274446	CC	319	448		1				
	CT	132	286		0.63 (0.49, 0.83)		**<0.001**		**0.005**
	TT	22	41		0.72 (0.40, 1.30)		0.278		0.548
	Dominant model				0.65 (0.50, 0.83)		**<0.001**		**0.005**
	Recessive model				0.85 (0.48, 1.50)		0.568		0.705
	Additive model				0.72 (0.58, 0.89)		**0.003**		**0.016**
rs3748735	CC	376	573		1				
	CT	98	191		0.76 (0.57, 1.02)		0.068		0.179
	TT	7	14		0.89 (0.33, 2.38)		0.818		0.818
	Dominant model				0.77 (0.58, 1.02)		0.071		0.143
	Recessive model				0.95 (0.36, 2.54)		0.920		0.920
	Additive model				0.80 (0.62, 1.04)		0.096		0.143

OP, osteoporosis; OR, odds ratio; CI, confidence interval.

a Covariates included age, body mass index and age of menopause (for women only).

b P values corrected for false discovery rate.

Boldness indicates the results achieve statistical significance (P<0.05).

**Table 5:** Multivariate linear regression results of associations between common genetic variants and BMDs in the combination dataset analysis.

SNPs	Models	LS BMD (g/cm^2^)			FN BMD (g/cm^2^)		
		β (95%CI)	*P* ^a^	*P*_FDR_ ^a^ ^b^	β (95%CI)	*P* ^a^	*P*_FDR_ ^a^ ^b^
rs2070296	Dominant model	-0.010 (-0.030, 0.010)	0.357	0.596	-0.003 (-0.020, 0.010)	0.725	0.725
	Recessive model	-0.030 (-0.050, -0.010)	**0.009**	0.053	-0.030 (-0.050, -0.010)	**<0.001**	**0.004**
	Additive model	-0.020 (-0.030, -0.001)	**0.033**	0.099	-0.010 (-0.020, -0.001)	**0.026**	0.077
rs4679323	Dominant model	-0.010 (-0.020, 0.030)	0.537	0.644	0.010 (-0.010, 0.020)	0.299	0.725
	Recessive model	-0.003 (-0.030, 0.020)	0.774	0.774	0.003 (-0.010, 0.020)	0.697	0.956
	Additive model	-0.001 (-0.010, 0.020)	0.836	0.894	0.004 (-0.010, 0.010)	0.380	0.576
rs73861745	Dominant model	0.010 (-0.010, 0.030)	0.397	0.596	0.004 (-0.010, 0.020)	0.574	0.725
	Recessive model	0.020 (-0.020, 0.050)	0.361	0.673	-0.001 (-0.030, 0.030)	0.956	0.956
	Additive model	0.010 (-0.010, 0.020)	0.295	0.59	0.002 (-0.010, 0.010)	0.670	0.670
rs1664227	Dominant model	-0.020 (-0.040, -0.001)	**0.039**	0.233	-0.010 (-0.020, 0.010)	0.525	0.725
	Recessive model	-0.030 (-0.050, -0.002)	**0.034**	0.102	-0.010 (-0.020, 0.010)	0.424	0.956
	Additive model	-0.020 (-0.030, -0.004)	**0.010**	0.063	-0.004 (-0.010, 0.010)	0.384	0.576
rs2274446	Dominant model	0.010 (-0.010, 0.030)	0.253	0.596	0.030 (0.010, 0.040)	**<0.001**	**0.002**
	Recessive model	-0.020 (-0.060, 0.030)	0.454	0.673	0.004 (-0.030, 0.040)	0.784	0.956
	Additive model	0.010 (-0.010, 0.020)	0.506	0.759	0.020 (0.010, 0.030)	**0.002**	**0.013**
rs3748735	Dominant model	-0.0003 (-0.020, 0.020)	0.980	0.980	0.004 (-0.010, 0.020)	0.608	0.725
	Recessive model	-0.020 (-0.090, 0.050)	0.561	0.673	0.003 (-0.050, 0.060)	0.922	0.956
	Additive model	-0.004 (-0.020, 0.020)	0.894	0.894	0.004 (-0.010, 0.020)	0.624	0.670

LS, lumbar spine; BMD, bone mineral density; FN, femoral neck; β, regression coefficient; CI, confidence interval.

a Covariates included age, body mass index and age of menopause (for women only).

b P values corrected for false discovery rate.

Boldness indicates the results achieve statistical significance (P<0.05).

**Table 6:** Multivariate linear regression results of associations between rare genetic variants and BMDs in the combination data analysis.

SNPs	LS BMD (g/cm^2^)		FN BMD (g/cm^2^)	
	β (95% CI)	*P* ^a^/*P*_FDR_ ^a^ ^b^	β (95% CI)	*P* ^a^/*P*_FDR_ ^a^ ^b^
rs180868035	-0.17 (-0.30, -0.05)	**0.008**/ **0.032**	-0.12 (-0.21, -0.03)	**0.010**/ **0.040**
rs369477952	-0.21 (-0.49, 0.07)	0.138/ 0.142	-0.19 (-0.39, 0.01)	0.058/ 0.116
rs146550621	-0.30 (-0.69, 0.10)	0.141/ 0.142	-0.20 (-0.48, 0.08)	0.170/ 0.227
rs767142032	-0.30 (-0.69, 0.10)	0.142/ 0.142	-0.12 (-0.40, 0.16)	0.383/ 0.383

LS, lumbar spine; FN, femoral neck; BMD, bone mineral density; β, regression coefficient; CI, confidence interval.

a Covariates included age, body mass index and age of menopause (for women only).

b P values corrected for false discovery rate.

Boldness indicates the results achieve statistical significance (P<0.05).

### 3.5 Effect of *NRP1* rs180868035 mutation on osteoblast and osteoclast differentiation

Considering our findings from above work and the bioinformatics analysis of function annotations, the *NRP1* rs180868035 was preferentially selected for function research *via in vitro* experiments. The *NRP1* rs180868035 is a nonsynonymous mutation located in the exonic region. It was predicted by the SIFT and Mutation Taster software that the *NRP1* rs180868035 T>G mutation can cause the coded amino acid (p.I140L) change (**Table S4**).

The ALP staining was positive in osteoblasts transfected with an empty vector, and vectors with either the wild-type (allele T at rs180868035, I140) or mutant (allele G at rs180868035, L140) *NRP1* gene sequence. Compared to the osteoblasts transfected with empty vector, wild-type transfected osteoblasts apparently enhanced the depth of ALP staining (**Figure S1**), as well as mRNA expression of the osteoblast marker genes, i.e., *ALP*, *RUNX2*, *SP7* and *OCN* (**Figure S2**). Moreover, compared to the wild-type transfected cells, the depth of ALP staining was apparently reduced (**Figure S1**), and osteoblast marker genes all were lower in the mutant transfected cells (**Figure S2**).

The TRAP staining was positive in osteoclasts transfected with an empty vector, and vectors with either the wild-type or mutant *NRP1* gene sequence. Compared to the osteoclasts transfected with empty vector (**Figure S3**), the depth of TRAP staining and osteoclast marker genes expression level, i.e., *TRAP*, *NFATc1* and *CTSK*, all were reduced in the wild-type transfected osteoclasts (**Figure S4**). Besides, compared to the wild-type transfected cells, mutant transfection significantly enhanced the depth of TRAP staining (**Figure S3**), and mRNA expression level of osteoclast marker genes (**Figure S4**).

### 3.6 Effect of *NRP1* rs180868035 mutation on osteoblast and osteoclast proliferation

**Figure 2:**
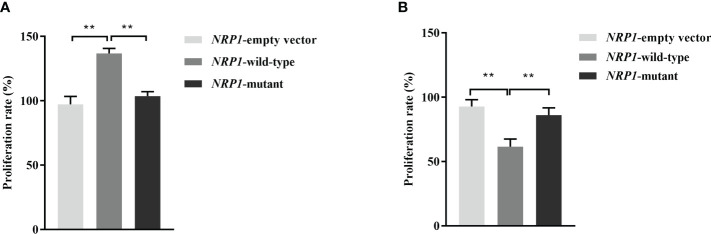
Proliferation rate comparisons among osteoblasts **(A)** and osteoclasts **(B)** transfected with empty and wild-type and mutant of *NRP1* rs180868035 vectors. ** indicated *P* < 0.001.

The average proliferation rates of osteoblasts transfected an empty vector, and vectors with either the wild-type and mutant *NRP1* were 97.3%, 136.8% and 103.5%, respectively. As shown in **Figure 2A**, compared to those transfected with the empty vector, the proliferation rate was significantly promoted in osteoblasts transfected with the wild-type *NRP1*; and compared to those transfected with the wild-type, the proliferation rate was significantly inhibited in osteoblasts transfected with the mutant *NRP1*.

The average proliferation rates of osteoclasts transfected an empty vector, and vectors with either the wild-type and mutant *NRP1* were 92.8%, 61.5% and 86.1%, respectively. As shown in **Figure 2B**, compared to those transfected with the empty vector, the proliferation rate was significantly inhibited in osteoclasts transfected with the wild-type *NRP1*; and compared to those transfected with the wild-type, the proliferation rate was significantly promoted in osteoclasts transfected with the mutant *NRP1*.

### 3.7 Effect of *NRP1* rs180868035 mutation on osteoblast and osteoclast apoptosis

**Figure 3:**
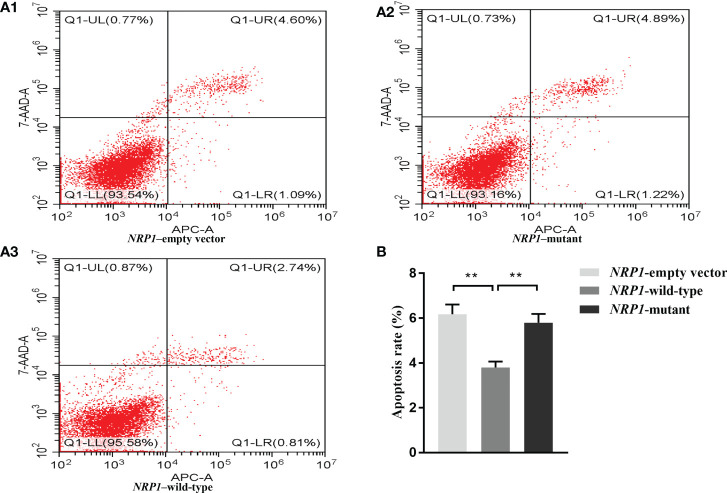
Cellular apoptosis comparisons among osteoblasts transfected with empty, and wild-type and mutant of *NRP1* rs180868035 vectors. **(A)** flow cytometry. Representative APC-A-area versus 7-AAD-A-area density plots of different treatment groups with percentages of cells at different stage of apoptosis shown in the relevant quadrants. Q1-UR quadrant is positive for both APC-A and 7-AAD-A and represents cells in late apoptosis; Q1-LR is APC-A positive/7-AAD-A negative and represents cells in early apoptosis; Q1-LL is negative for both APC-A and 7-AAD-A and contains viable cells; Q1-UL is 7-AAD-A positive/APC-A negative and contains cells undergoing necrosis. **(B)** histogram of osteoblast apoptosis. ** indicated *P* < 0.001.

The average apoptosis rates of osteoblasts transfected with an empty vector, and vectors with either the wild-type and mutant *NRP1* were 6.2%, 3.8% and 5.8%, respectively. Compared to those transfected with the empty vector, the apoptosis rate was significantly reduced in osteoblasts transfected with the wild-type *NRP1*; and compared to those transfected with the wild-type, the apoptosis rate was significantly increased in osteoblasts transfected with the mutant *NRP1* (**Figure 3**).

**Figure 4:**
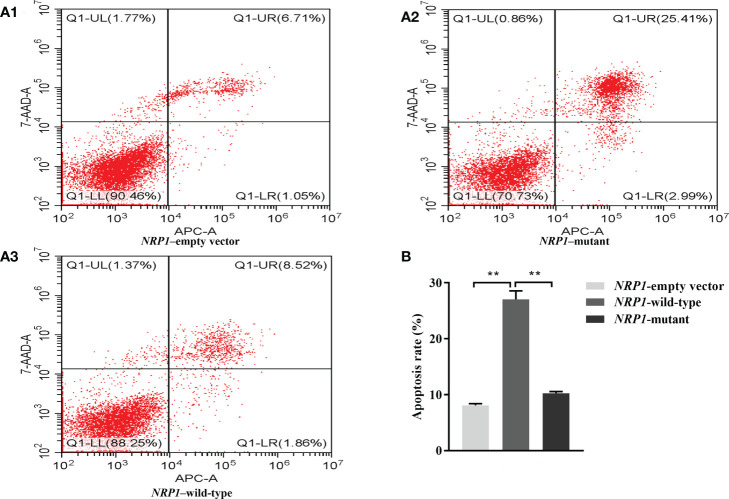
Cellular apoptosis comparisons among osteoclasts transfected with empty, and wild-type and mutant of *NRP1* rs180868035 vectors. **(A)** flow cytometry. Representative APC-A-area versus 7-AAD-A-area density plots of different treatment groups with percentages of cells at different stage of apoptosis shown in the relevant quadrants. Q1-UR quadrant is positive for both APC-A and 7-AAD-A and represents cells in late apoptosis; Q1-LR is APC-A positive/7-AAD-A negative and represents cells in early apoptosis; Q1-LL is negative for both APC-A and 7-AAD-A and contains viable cells; Q1-UL is 7-AAD-A positive/APC-A negative and contains cells undergoing necrosis. **(B)** histogram of osteoclast apoptosis. ** indicated *P* < 0.001.

The average apoptosis rates of osteoclasts transfected with an empty vector, and vectors with either the wild-type and mutant *NRP1* were 8.1%, 27.0% and 10.2%, separately. Compared to those transfected with the empty vector, the apoptosis rate was significantly elevated in osteoclasts transfected with the wild-type *NRP1*; and compared to those transfected with the wild-type, the apoptosis rate was significantly decreased in osteoclasts transfected with the mutant *NRP1* (**Figure 4**).

## 4 Discussion

In the present study, we conducted a two-stage designed epidemiological research in combination with bioinformatics analysis to identify functional genetic variants associated with OP risk/BMD in 5 SEMA3A pathway genes (*SEMA3A*, *NRP1*, *PLXNA1*, *PLXNA2* and *PLXNA3*). *In vitro* experiments were further performed to investigate roles of identified variants in bone mass remodeling. The *NRP1* rs2070296 and rs180868035, and *PLXNA2* rs2274446 were revealed to be associated with OP risk/BMD. Based on function annotations to those variants, *in vitro* experiments were conducted and showed that the *NRP1* rs180868035 mutation affect differentiation, proliferation and apoptosis of osteoblasts and osteoclasts.

Our findings showed that the *NRP1* rs2070296 TT genotype was associated with increased OP risk and decreased FN BMD, and LS and FN BMDs decreased in response to the *NRP1* rs180868035 G allele mutation. The rs2070296 C>T (V179V) is a synonymous mutation at physical position of chr10:33552695, located in exon 4 of the *NRP1* gene. According to the rSNPBase database (http://rsnp3.psych.ac.cn/index.do), the rs2070296 is either a regulatory SNP or an expression quantitative trait locus, indicating it may be involved in the regulation of *NRP1* gene expression. We found little evidence on the association of the rs2070296 variant with specific outcomes other than one literature observed a cumulative effect of SNP rs2070296 on visual acuity underwent ranibizumab treatment (20). The rs180868035 T>G (I140L) is a nonsynonymous mutation at physical position of chr10:33559615 located in exon 3 of the *NRP1* gene, corresponding to the change of Ile amino acid to Leu amino acid (p.I140L). It was predicted by the SIFT algorithm to be deleterious to protein function (21). No study on the association between genetic variant of *NRP1* rs180868035 and bone metabolism was reported before ours.

We further performed *in vitro* experiments to explore the influence of *NRP1* mutation on bone metabolism. Our findings revealed that overexpression of a rs180868035 T>G mutant decreased the expression levels of osteoblast marker genes, i.e., *ALP* (22), *RUNX2* (23), *SP7* (24) and *OCN* (25), inhibited osteoblast proliferation and promoted osteoblast apoptosis. In the meanwhile, the overexpression of the mutant was found to enhance the expression of osteoclast marker genes, i.e., *TRAP* (26), *NFATc1* and *CTSK* (27), promote osteoclast proliferation and inhibit osteoclast apoptosis. Findings from *in vitro* experiments corroborated the association of *NRP1* rs180868035 with bone phenotype found in our epidemiological study. The N-terminal of SEMA3A contains a seven-bladed β-propeller structure, which was reckoned as the signature ‘Sema’ domain, determining its unique ability to bind to NRP1, and the *NRP1* rs180868035 genetic variant is located in the functional domain in extracellular region of *NRP1* that binds to the SEMA3A (28, 29). The *Npn1*-mutant mice (NRP1^Sema-^ mice), lacking the Sema-binding site, exhibited a same osteoporotic phenotype as *SEMA3A*-deficient mice, indicating that the NRP1 functions through binding to SEMA3A (13). In addition, the binding of SEMA3A to NRP1 could inhibit osteoclast differentiation induced by RANKL signaling and enhance osteoblast differentiation through classic WNT/β catenin pathway (9, 13). We therefore speculated that the *NRP1* mutation caused amino acid sequence alteration that resulted in the inability of NRP1 to bind to SEMA3A, further disrupting the differentiation, proliferation and apoptosis of osteoblasts and osteoclasts. The NRP1 served as a co-receptor and interacted with the discoidin domain receptor 2 (DDR2)—a receptor tyrosine kinase possessing a role of promoting bone-depositing cells and osteoblasts differentiation. Animal experiments suggested that NRP1 enhanced the bone formation induced by DDR2, and knockdown of *NRP1* or ectopic expression of *NRP1* can influence osteoclastogenesis in cells with different DDR2 expression levels (30). Thus, *NRP1* mutation might inhibit the interaction between NRP1 and DDR2. It is an intriguing idea for the future studies. Besides, NRP1 expression was tightly regulated by growth factors, transcription factors, and injury, e.g., tumor necrosis factor-alpha, Prox-1, and ischemia (31). But evidence on how these modulators regulate NRP1 in the process of OP development is still scarce. More studies are needed to investigate potential mechanisms.

We found genetic variant of *PLXNA2* rs2274446 (C > T) was associated with reduced OP risk and increased FN BMD. PLXNA2 is one of transmembrane receptors and can mediate SEMA3A signals through binding to NRP1 as a co-receptor for SMEA3A (32). Oh et al. found that the *PLXNA2* expression could be upregulated by the bone morphogenic protein 2, subsequently activating the expression of two major osteogenic transcription factors, Runx2 and SP7, and reinforcing osteogenic mineralization and differentiation, indicating that the *PLXNA2* possesses potential ability to affect bone remodeling and is a noteworthy target for OP treatment (18). PLXNA2 is a receptor of SEMA6A in osteoclasts. The SEMA6A-PLXNA2 axis participated in osteoclastogenesis induced by RANKL, and this function is dependent on the activation of NFATc1 induced by PLCγ (33). It offers potential therapeutic targets in the intervention of OP. In a study including 560 postmenopausal women of Korean ethnicity, ten SNPs in the *PLXNA2* gene were selected for genotyping and the rs3748735 variant was found to be significantly associated with FN BMD (32). Genetic variant of *PLXNA2* rs3748735 was found to be associated with FN BMD at the discovery stage in our study, while the significant association did not maintain at the further validation stages. This may be partly due to different sample sizes and study populations.

As far as we known, our study is one of few studies to explore the association of genetic variants in the SEMA3A signaling pathway genes with OP risk. Our work consisted of population-based epidemiological study and *in vitro* experiments, concluding genetic variant of *NRP1* rs180868035 may affect BMD by increasing bone resorption and reducing bone formation. Results from these two parts corroborate and complement each other. Thus, our study can provide stable and valid evidence for OP susceptibility loci identification. Meanwhile, several limitations should be mentioned. First, though we found genetic variant of *NRP1* rs180868035 could affect osteoblast and osteoclast proliferation and apoptosis, the specific biological mechanism through which *NRP1* rs180868035 influence bone metabolism needs to be elucidated in the future. Second, the extensibility of our findings will be limited because we only included Chinese Han residents living in Wuhan, China. Other population-based studies are needed to confirm our findings. Third, in the *in vitro* experiment part, the early stage of osteoblasts and osteoclasts differentiation was missed to be measured in this study. At last, several potential confounders, e.g., physical activity (34), serum calcium and vitamin D levels (35), were not adjusted in the present study due to insufficient data, which would bias the conclusions.

## 5 Conclusion

This study firstly and systematically analyzed the associations between genetic variants in the SEMA3A signaling pathway genes and OP risk/BMD in a Chinese population. We concluded genetic variants of *NRP1* rs2070296 and rs180868035, and *PLXNA2* rs2274446 were associated with OP risk and BMDs. The *NRP1* rs180868035 influences bone metabolism by regulating osteoblast and osteoclast differentiation, proliferation and apoptosis. More researches are needed to prove our findings.

## Data availability statement

The datasets presented in this study can be found in online repositories. The names of the repository/repositories and accession number(s) can be found below: www.ncbi.nlm.nih.gov/bioproject/, PRJNA884817.

## Ethics statement

The studies involving human participants were reviewed and approved by The Ethics Committee of Tongji Medical College of Huazhong University of Science and Technology. The patients/participants provided their written informed consent to participate in this study.

## Author contributions

Conceptualization: QW, QH, H-LZ and M-HW. Study conduct: H-LZ and M-HW. Data collection and preparation: D-SD, R-YZ, J-LZ, T-TY, QL and T-TZ. Data analysis: M-HW and H-LZ. Writing - Original draft preparation: H-LZ and M-HW. Wring – Review and Editing: QW and QH. All authors read, reviewed and approved the final manuscript. QW and QH had primary responsibility for final content. All authors contributed to the article and approved the submitted version.

## Funding

This work was supported by the National Natural Science Foundation of China (Grant nos.82273711 and 72061137006) and Wuhan Municipal Health Commission (Grant nos. WY22B06 and WG20C09).

## Acknowledgments

We would like to thank the staff of the two communities in Wuhan and physical examination and rehabilitation center of Wuhan Union hospital for their administrative and technology assistance.

## Conflict of interest

The authors declare that the research was conducted in the absence of any commercial or financial relationships that could be construed as a potential conflict of interest.

## Publisher’s note

All claims expressed in this article are solely those of the authors and do not necessarily represent those of their affiliated organizations, or those of the publisher, the editors and the reviewers. Any product that may be evaluated in this article, or claim that may be made by its manufacturer, is not guaranteed or endorsed by the publisher.

## Abbreviations

ALP, alkaline phosphatase; BMD, bone mineral density; BMI, body mass index; CTSK, cathepsin K; DXA, dual-energy X-ray absorptiometry; FDR, false discovery rate; FN, femoral neck; HWE, Hardy-Weinberg equilibrium; iMLDR, improved multiple linkage detection reaction; IQR, interquartile range; LS, lumbar spine; MAF, minor allele frequency; NFATc1, nuclear factor of acid phosphatase; NGS, next generation sequencing; NRP1, neuropilin 1; OCN, osteocalcin; OD, optical density; OP, osteoporosis; PLXNA1, plexinA1; PLXNA2, plexinA2; PLXNA3, plexinA3; SD, standard deviation; SEMA3A, semaphoring 3A; SIFT, sorting intolerant from tolerant; SNP, single nucleotide polymorphisms; TRAP, tartrate-resistant acid phosphatase.
