# Supplementary figures and images for: Association between SEMA3A signaling pathway genes and BMD/OP risk: An epidemiological and experimental study
Source: Front Endocrinol (Lausanne). 2022 Nov 8;13:1014431. doi: 10.3389/fendo.2022.1014431 (PMC9679019; doi:10.3389/fendo.2022.1014431)

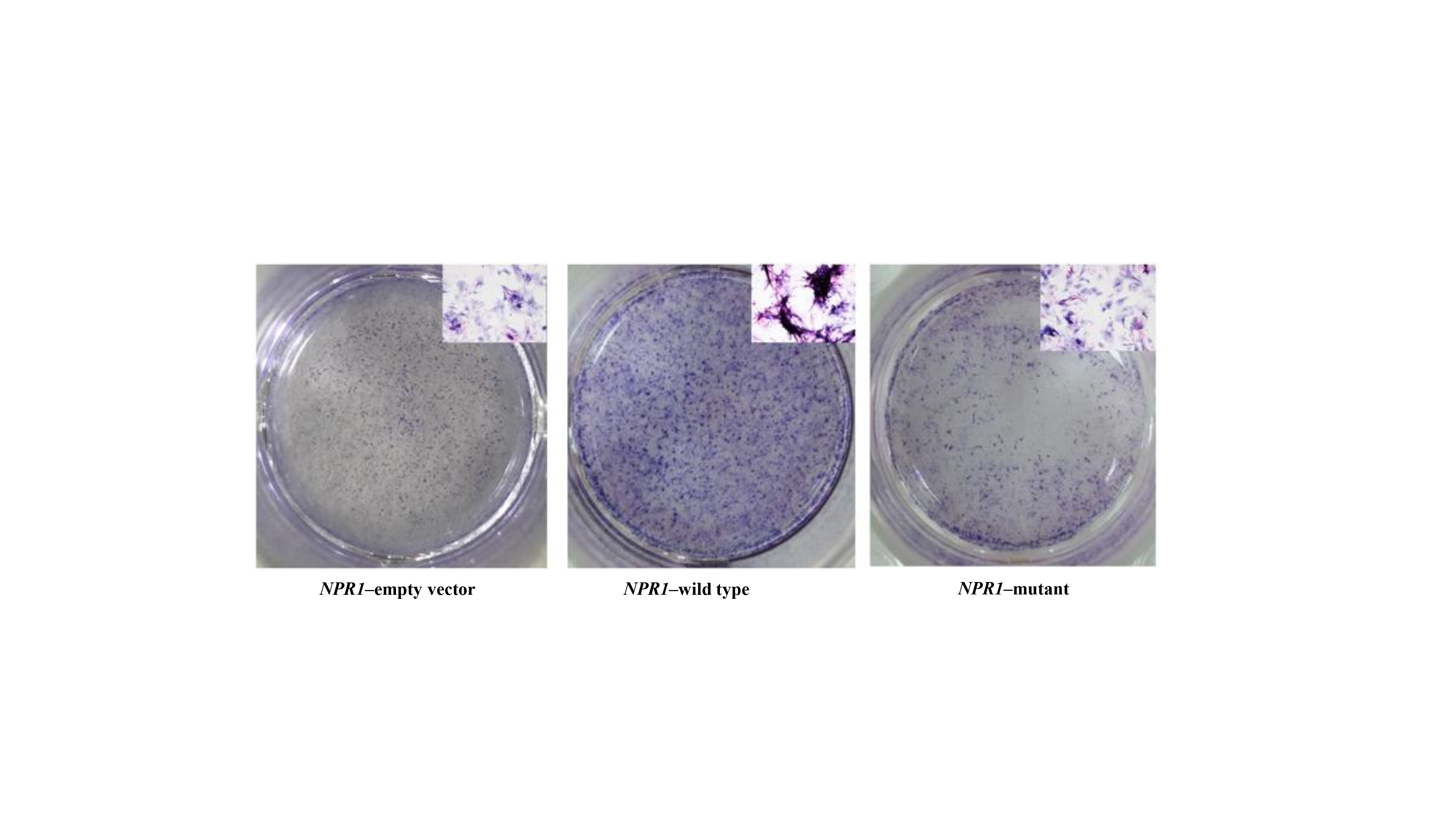

Supplement: Supplementary file 1 [file Image_1.tif]

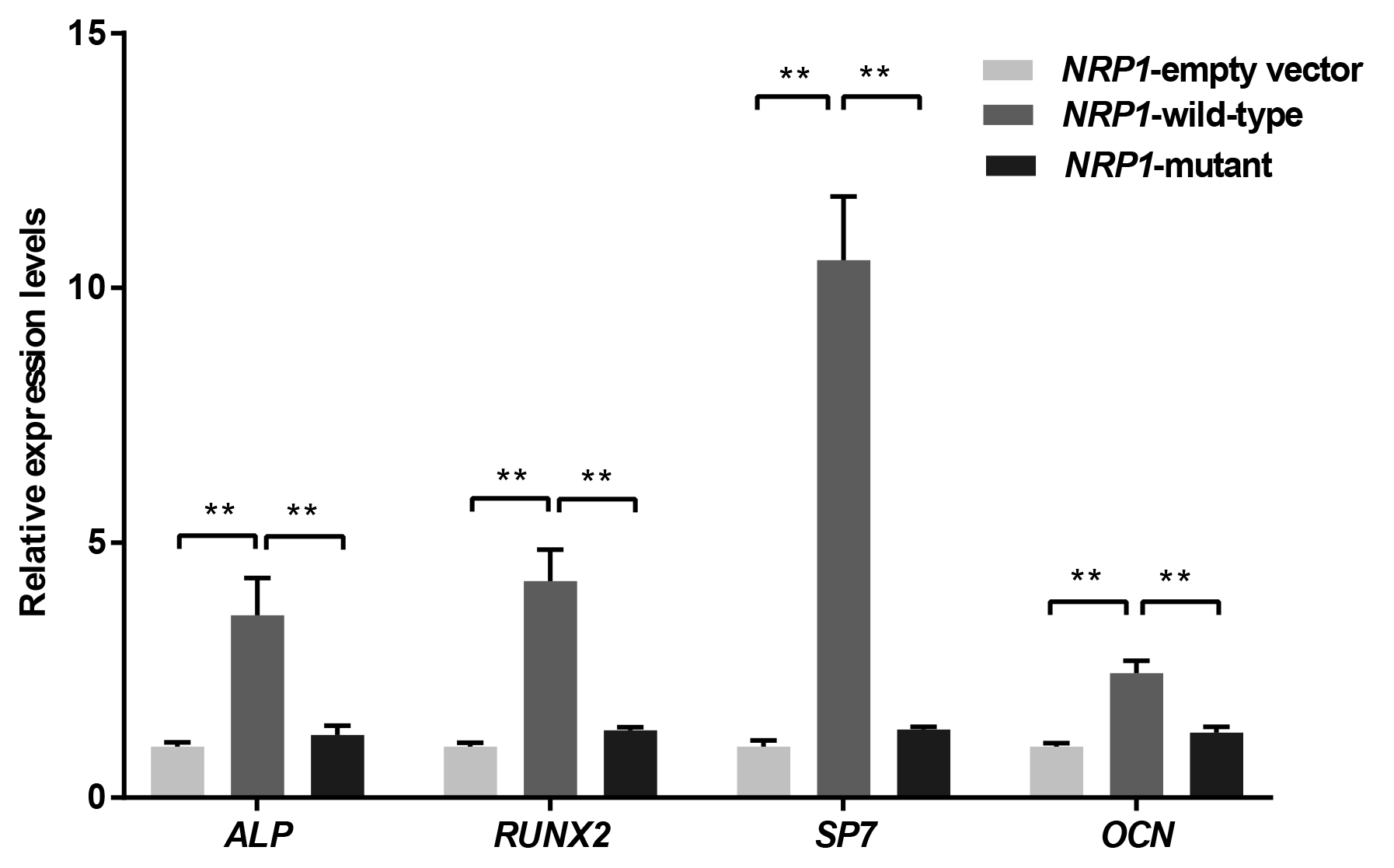

Supplement: Supplementary file 2 [file Image_2.tif]

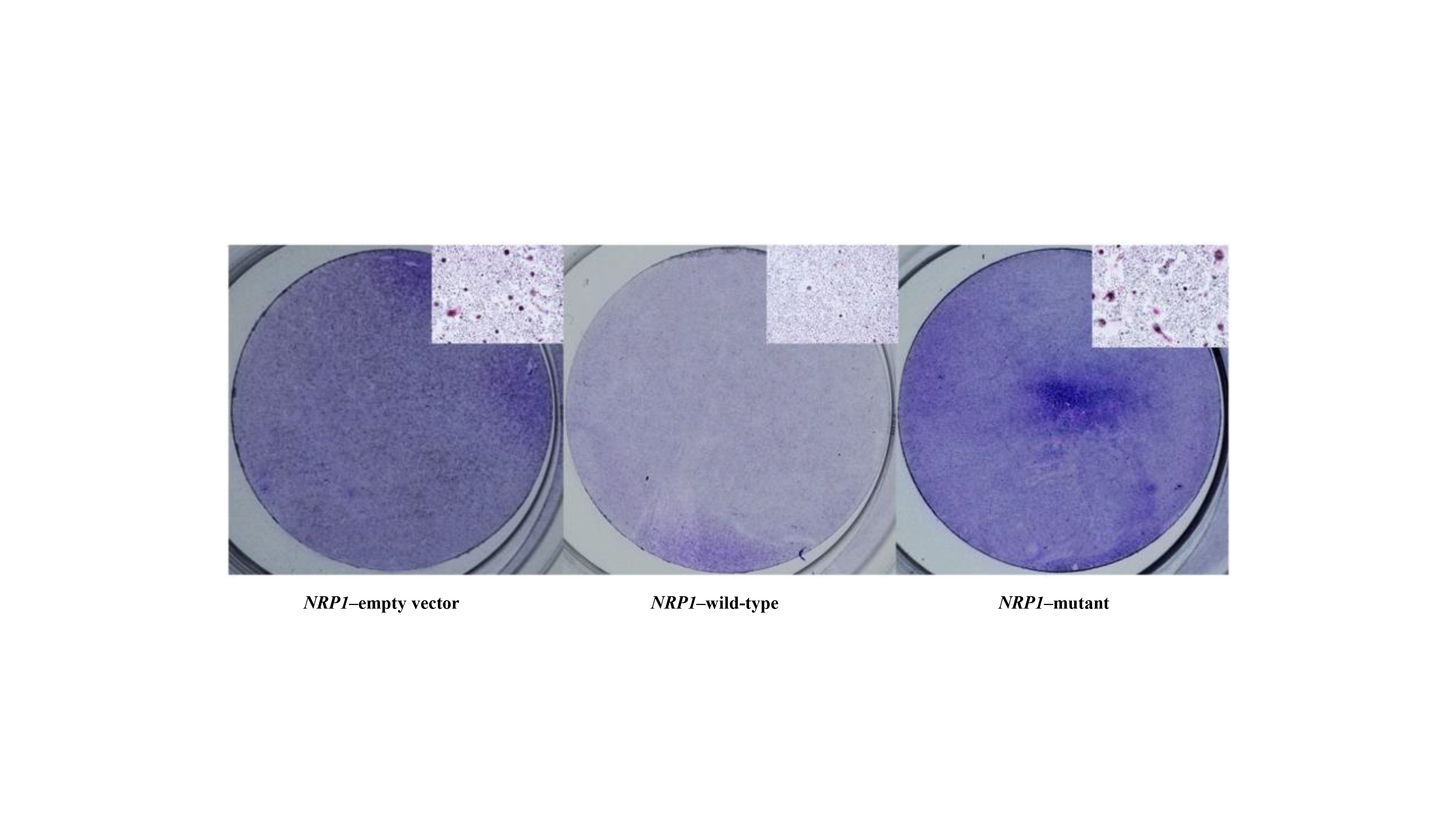

Supplement: Supplementary file 3 [file Image_3.tif]

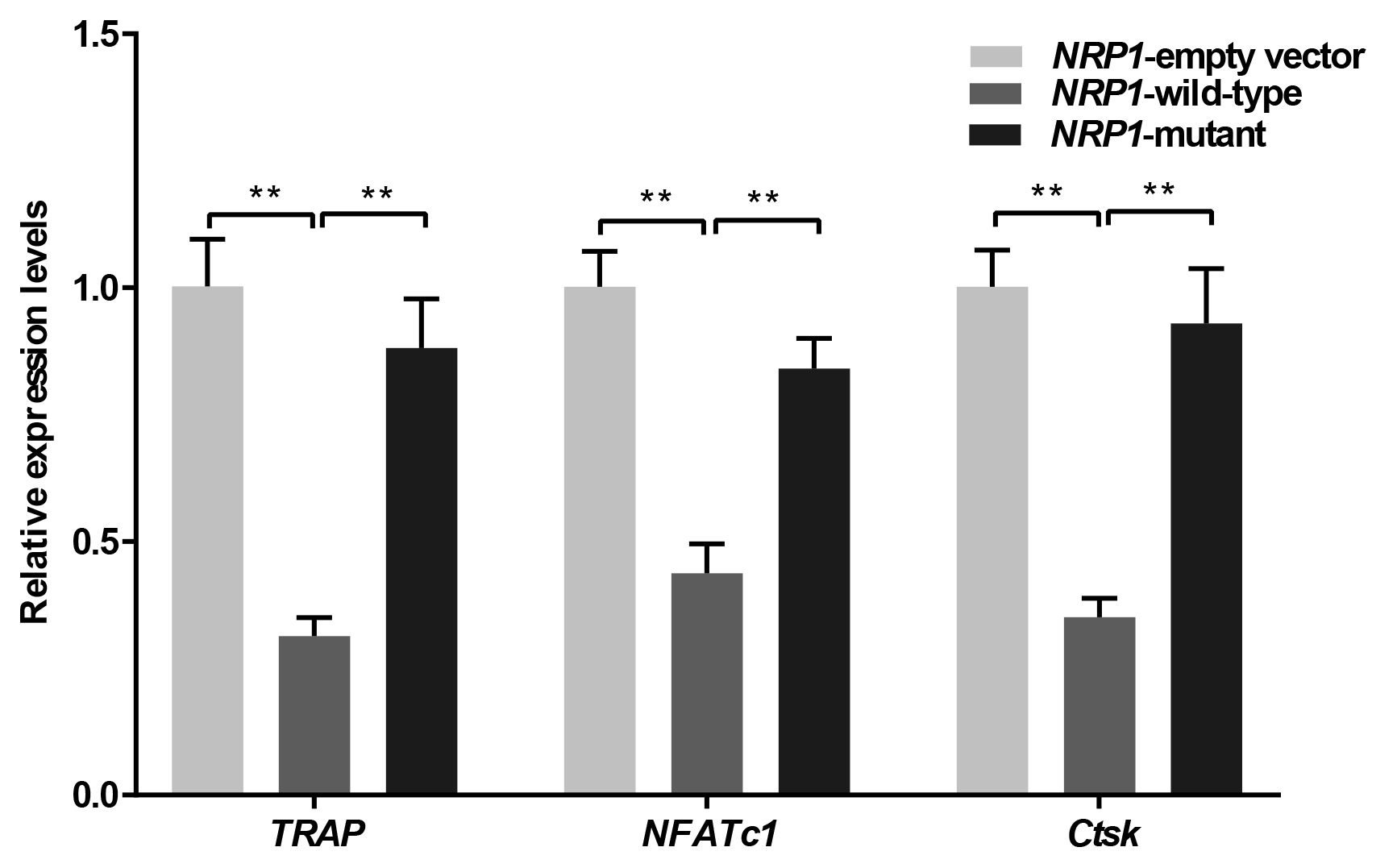

Supplement: Supplementary file 4 [file Image_4.tif]
