# Supplementary material for: Association between SEMA3A signaling pathway genes and BMD/OP risk: An epidemiological and experimental study
Source: Front Endocrinol (Lausanne). 2022 Nov 8;13:1014431. doi: 10.3389/fendo.2022.1014431 (PMC9679019; doi:10.3389/fendo.2022.1014431)
Supplement: Supplementary file 5 [file Table_1.docx]

Table S1. Characteristics of newly discovered non-synonymous mutation in the discovery stage.

| Gene | Chr | Location | Ref | Alt | Change of amino acid | SIFT | POLYPhen2 | Mutation Taster |
| --- | --- | --- | --- | --- | --- | --- | --- | --- |
|  |  |  |  |  |  | Function prediction score | | |
| *SEMA3A* | 7 | 83643633 | C | G | p.E234D | 1.000 | 0.394 | 1.000 |
| *SEMA3A* | 7 | 83590772 | C | T | p.S744N | 0.506 | 0 | 1.000 |
| *NRP1* | 10 | 33545300 | A | G | p.I253T | 0.037 | 0.999 | 1.000 |
| *PLXNA1* | 3 | 126736424 | C | G | p.L1145V | 0.755 | 0 | 0.710 |
| *PLXNA1* | 3 | 126707449 | C | T | p.P5S | 0 | - | 0.991 |
| *PLXNA2* | 1 | 208205052 | A | G | p.V1703A | 1.000 | 0.002 | 1.000 |
| *PLXNA1* | 3 | 126707470 | C | A | p.L12M | 0.122 | - | 1.000 |
| *PLXNA2* | 1 | 208391192 | C | T | p.V26M | 0.130 | 0.013 | 1.000 |
| *PLXNA2* | 1 | 208383731 | A | G | p.L422P | 0.327 | 0.033 | 1.000 |
| *PLXNA3* | X | 153693173 | G | A | p.E669K | 0.161 | 0.006 | 0.887 |
| *PLXNA3* | X | 153688827 | A | G | p.I102V | 0.254 | 0.005 | 0.993 |
| *PLXNA3* | X | 153693101 | A | T | p.M645L | 0.990 | 0 | 0.978 |
|  |  |  |  |  |  | Function prediction results | | |
| *SEMA3A* | 7 | 83643633 | C | G | p.E234D | tolerated | benign | disease causing |
| *SEMA3A* | 7 | 83590772 | C | T | p.S744N | tolerated | benign | polymorphism |
| *NRP1* | 10 | 33545300 | A | G | p.I253T | damaging | probably damaging | disease causing |
| *PLXNA1* | 3 | 126736424 | C | G | p.L1145V | tolerated | benign | disease causing |
| *PLXNA1* | 3 | 126707449 | C | T | p.P5S | damaging | - | disease causing |
| *PLXNA2* | 1 | 208205052 | A | G | p.V1703A | tolerated | benign | disease causing |
| *PLXNA1* | 3 | 126707470 | C | A | p.L12M | tolerated | - | polymorphism |
| *PLXNA2* | 1 | 208391192 | C | T | p.V26M | tolerated | benign | polymorphism |
| *PLXNA2* | 1 | 208383731 | A | G | p.L422P | tolerated | benign | disease causing |
| *PLXNA3* | X | 153693173 | G | A | p.E669K | tolerated | benign | disease causing |
| *PLXNA3* | X | 153688827 | A | G | p.I102V | tolerated | benign | disease causing |
| *PLXNA3* | X | 153693101 | A | T | p.M645L | tolerated | benign | polymorphism |

Chr, chromosome; Ref, reference allele; Alt, mutant allele; SEMA3A, semaphorin 3A; NRP1, Neuropilin-1; PLXNA1, plexinA1; PLXNA2, plexinA2; PLXNA3, plexinA3;

Table S2. Multivariate logistic regression results of associations between common genetic variants and OP risk in the discovery stage.

| Rs number | Genotype | Case | Control | OR (95%CI) | *P*^a^ |
| --- | --- | --- | --- | --- | --- |
| rs2070296 | CC | 50 | 50 | 1 | - |
|  | CT | 97 | 104 | 0.81 (0.47, 1.39) | 0.443 |
|  | TT | 62 | 35 | 1.72 (0.90, 3.26) | 0.100 |
|  | Dominant model |  |  | 1.02 (0.62, 1.70) | 0.927 |
|  | Recessive model |  |  | 1.98 (1.16, 3.36) | **0.012** |
|  | Additive model |  |  | 1.29 (0.94, 1.78) | 0.110 |
| rs4679323 | CC | 63 | 44 | 1 | - |
|  | CA | 99 | 103 | 0.53 (0.31, 0.90) | **0.019** |
|  | AA | 43 | 42 | 0.68 (0.36, 1.28) | 0.236 |
|  | Dominant model |  |  | 0.57 (0.35, 0.94) | **0.029** |
|  | Recessive model |  |  | 1.03 (0.60, 1.74) | 0.925 |
|  | Additive model |  |  | 0.81 (0.59, 1.10) | 0.178 |
| rs73861745 | GG | 113 | 110 | 1 | - |
|  | GA | 69 | 69 | 0.86 (0.54, 1.39) | 0.545 |
|  | AA | 17 | 8 | 2.66 (0.99, 7.15) | 0.052 |
|  | Dominant model |  |  | 1.02 (0.65, 1.60) | 0.929 |
|  | Recessive model |  |  | 2.81 (1.06, 7.43) | **0.038** |
|  | Additive model |  |  | 1.18 (0.83, 1.70) | 0.356 |
| rs1664227 | GG | 52 | 67 | 1 | - |
|  | GC | 110 | 91 | 1.64 (0.99, 2.72) | 0.055 |
|  | CC | 47 | 31 | 2.04 (1.07, 3.86) | **0.029** |
|  | Dominant model |  |  | 1.74 (1.08, 2.81) | **0.023** |
|  | Recessive model |  |  | 1.49 (0.86, 2.60) | 0.157 |
|  | Additive model |  |  | 1.45 (1.06, 1.99) | **0.022** |
| rs2274446 | CC | 132 | 103 | 1 | - |
|  | CT | 59 | 78 | 0.55 (0.34, 0.88) | **0.012** |
|  | TT | 12 | 5 | 1.39 (0.41, 4.70) | 0.594 |
|  | Dominant model |  |  | 0.60 (0.38, 0.94) | **0.027** |
|  | Recessive model |  |  | 1.73 (0.52, 5.76) | 0.373 |
|  | Additive model |  |  | 0.73 (0.50, 1.07) | 0.110 |
| rs3748735 | CC | 169 | 136 | 1 | - |
|  | CT | 39 | 50 | 0.55 (0.32, 0.93) | **0.026** |
|  | TT | 3 | 3 | 1.10 (0.21, 5.79) | 0.913 |
|  | Dominant model |  |  | 0.58 (0.35, 0.96) | **0.035** |
|  | Recessive model |  |  | 1.25 (0.24, 6.59) | 0.794 |
|  | Additive model |  |  | 0.65 (0.41, 1.03) | 0.069 |

OP, osteoporosis; OR, odds ration; CI, confidence interval.

^a^ Covariates included age, body mass index and menopause age (for women only).

Boldness indicates the results achieve statistical significance.

Table S3. Distribution of the number of the 4 rare variants alleles in OP group, non-OP group and the East Asian population of the GnomAD database at the discovery stage.

| Genes | Rs number | Chr | Location | Ref | Alt | OP group | |  | Non-OP group | |  | GnomAD | | *P*^&^ | *P*^#^ |
| --- | --- | --- | --- | --- | --- | --- | --- | --- | --- | --- | --- | --- | --- | --- | --- |
|  |  |  |  |  |  | Allele count | Allele number |  | Allele count | Allele number |  | Allele count | Allele number |  |  |
| *NRP1* | rs180868035 | 10 | 33559615 | T | G | 4 | 420 |  | 3 | 392 |  | 45 | 19744 | 1 | 0.019 |
| *NRP1* | rs767142032 | 10 | 33469195 | T | C | 1 | 420 |  | 0 | 378 |  | 0 | 18394 | 1 | 0.022 |
| *PLXNA1* | rs369477952 | 3 | 126724930 | C | T | 2 | 418 |  | 0 | 378 |  | 3 | 18388 | 0.501 | 0.005 |
| *PLXNA1* | rs146550621 | 3 | 126736652 | G | A | 1 | 418 |  | 0 | 378 |  | 0 | 18342 | 1 | 0.022 |
| Chr, chromosome; Ref, reference allele; Alt, alter allele; SEMA3A, semaphorin 3A; NRP1, Neuropilin-1; PLXNA1, plexinA1; PLXNA2, plexinA2; PLXNA3, plexinA3; &, Fisher’s exact test was adopted to compare the allele frequencies of OP and non-OP group; #, the East Asian population of the GnomAD database was used as external control, and Fisher’s exact test was used to compare the allele frequencies of OP and external control group. | | | | | | | | | | | | | | | |
|  | | | | | | | | | | | | | | | |

Table S4. Characteristics of the 10 OP-related genetic variants discovered in the discovery stage.

| Gene | Rs number | Chr | Location | Ref | Alt | Region | Function | Change of amino acid | SIFT | POLYPhen2 | Mutation Taster |
| --- | --- | --- | --- | --- | --- | --- | --- | --- | --- | --- | --- |
| *NRP1* | rs2070296 | 10 | 33552695 | C | T | exonic | synonymous | p.V179V |  |  |  |
| *NRP1* | rs180868035 | 10 | 33559615 | T | G | exonic | nonsynonymous | p.I140L | D:0.003 | B:0.010 | D:0.999 |
| *NRP1* | rs767142032 | 10 | 33469195 | T | C | exonic | nonsynonymous | p.I861V | D:0 | D:0.998 | D:1 |
| *PLXNA1* | rs4679323 | 3 | 126737268 | C | A | exonic | synonymous | p.L1264L |  |  |  |
| *PLXNA1* | rs73861745 | 3 | 126737466 | G | A | intronic |  |  |  |  |  |
| *PLXNA1* | rs369477952 | 3 | 126724930 | C | T | exonic | nonsynonymous | p.R636W | T:0.136 | D:0.999 | D:0.999 |
| *PLXNA1* | rs146550621 | 3 | 126736652 | G | A | exonic | nonsynonymous | p.G1193S | D:0 | D:0.999 | D:1 |
| *PLXNA2* | rs1664227 | 1 | 208390086 | G | C | exonic | synonymous | p.T394T |  |  |  |
| *PLXNA2* | rs2274446 | 1 | 208252456 | C | T | intronic |  |  |  |  |  |
| *PLXNA2* | rs3748735 | 1 | 208390469 | C | T | exonic | nonsynonymous | p.A267T | T:0.096 | P:0.649 | P:0.015 |

Chr, chromosome; Ref, reference allele; Alt, mutant allele; SEMA3A, semaphorin 3A; NRP1, Neuropilin-1; PLXNA1, plexinA1; PLXNA2, plexinA2. SIFT: “T” means “tolerated”, “D” means “damaging”. PolyPhen2: “B” means “benign”, “P” means “possibly damaging”, “D” means “probably damaging”; Mutation Taster: “D” means “disease causing”, “P” means “polymorphism_automatic”.
